# CLINICAL RESULTS OF COLLAGENASE TREATMENT FOR DUPUYTREN’S DISEASE: A CASE SERIES STUDY WITH 2-YEARS FOLLOW-UP

**DOI:** 10.1590/1413-785220233101e259218

**Published:** 2023-04-17

**Authors:** Morena Anna Basso, Alessio Bernasconi, Giovanni Balato, Andrea Cozzolino, Giulia Famiglietti, Francesco Smeraglia

**Affiliations:** 1 “Federico II” University, Department of Public Health, Division of Orthopaedic Surgery, Naples, Italy.

**Keywords:** Dupuytren Contracture, Palmar Fibromatosis, Microbial Collagenase, Recurrence, Contratura de Dupuytren, Fibromatose Palmar, Colagenase Microbiana, Recidiva

## Abstract

**Objectives:**

This study aims to report our experience with Clostridium Histolyticum collagenase (CCH) to support the importance of its clinical use and assess its clinical efficacy, complications, and recurrences.

**Methods:**

This prospective observational study of 66 patients with a 2-year follow-up. Patients with an extension lag major of 20° at the metacarpophalangeal joint (MPJ) and/or proximal interphalangeal joint (PIPJ) were included. We collected data on demographic and anamnestic details, MPJ and PIPJ contracture degrees, DASH score, complications, and recurrences.

**Results:**

The mean pre-injection contracture was 34° for MPJ and 31° for PIPJ. At the 2-year follow-up, the mean contracture for the MPJ and PIPJ were respectively 3° and 14.5°. The mean DASH score decreased from 21.8 before injection to 10,4 after 2 years. The disease recurrence occurred in 34.8% of the patients, all with PIPJ contracture. The main complication was skin breakage (25.7%).

**Conclusion:**

The CCH injections remain a consistent option in treating DD; withdrawal from the European market deprives surgeons and patients of low invasiveness and safe tool for treating DD. ***Level of evidence IV, Therapeutic study investigating treatment results, Case series***.

## RESUMO

**Objetivos:**

O objetivo deste estudo é relatar nossa experiência com Clostridium Histolyticum colagenase (CCH) para apoiar a importância de seu uso clínico e para avaliar sua eficácia clínica, complicações e recidivas.

**Métodos:**

Estudo observacional prospectivo de acompanhamento por 2 anos em 66 pacientes com um atraso de extensão maior de 20° na articulação metacarpofalângica (MPJ) e/ou articulação interfalângica proximal (PIPJ). Foram coletados dados sobre detalhes demográficos e anamnésicos, graus de contração da MPJ e PIPJ, escore de DASH, complicações e recidivas.

**Resultados:**

A média da contração pré-injeção foi de 34° para a MPJ e 31° para a PIPJ. Com 2 anos de acompanhamento, a contração média para a MPJ e PIPJ foi de 3° e 14,5° respectivamente. A pontuação média do DASH diminuiu de 21,8 antes da injeção para 10,4 após 2 anos. A recorrência da doença ocorreu em 34,8% dos pacientes, todos com contração de PIPJ. A principal complicação foi a quebra da pele (25,7%).

**Conclusão:**

As injeções de CCH continuam sendo uma opção consistente no tratamento do DD; a retirada do medicamento do mercado europeu priva os cirurgiões e pacientes de uma ferramenta pouco invasiva e segura para o tratamento do DD. ***Nível de evidência IV, Estudo terapêutico que investiga os resultados do tratamento, série de casos***.

## INTRODUCTION

Dupuytren's disease (DD) is a chronic connective disorder of the palmar fascia of the hand. Usually the disease begins as a palpable nodule and then evolves to form cords with progressive contracture in flexion of the finger.^1^ When the fingers flex into the palm, the daily life is severely impaired.^2^

The prevalence of the disease is 0.2% but it can raise up to 50% in some subgroups of patients.^3^ Surgical treatments such as open fasciectomy or needle fasciotomy have always been the gold standard in the treatment of DD, but several complications can occur.^4^ In this context enzyme fasciotomy represents an attractive solution, so it has been studied and in the 1996^5^ Clostridium Histolyticum collagenase (CCH) was tested in vitro, this enzyme had the advantage to be collagen-specific. The first clinical study was published in 2000^6^ and in 2010 The Food and Drug Administration approved the collagenase clostridium histolyticum for the management of DD. In January 2020 CCH was withdrawn from European Union for commercial reasons.^7^ In this contest we want to report our clinical experience with CCH to support the importance of the clinical use of this useful drug, which re-defined the gold standard in the treatment of DD.^8^

## MATERIALS AND METHODS

**Figure 1:**
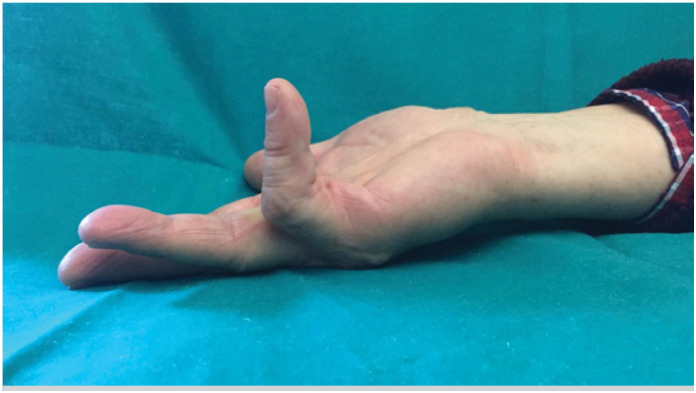
Pre-injection contracture of the V digit.

**Figure 2:**
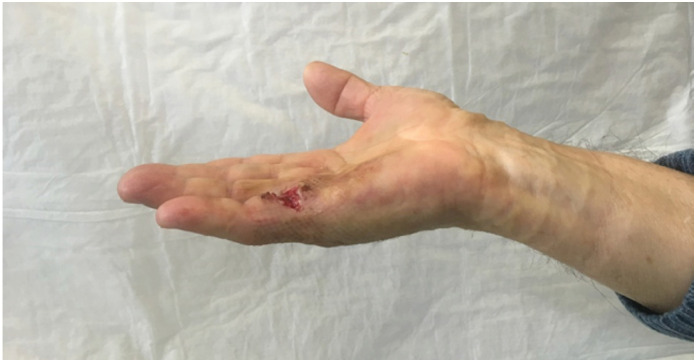
Contracture correction after the rupture of the cord.

This study is a prospective observational study which started in January 2018. We performed at our institution 66 CCH injections on 66 patients. All the patients expressed written consent. Our institution does not require an ethical approval for observational study. The study is in accordance with Helsinki criteria. The aim of the study is to assess the clinical efficacy of the CCH in the treatment of DD and its complications and recurrences. We included patients with a palpable cord and an extension lag major of 20° at metacarpophalangeal joint (MPJ) and/or proximal interphalangeal joint (PIPJ) were included in the study (Figure 1). We excluded from the study pregnant women, and patient with a Tubiana classification of IV. The injection procedure was performed in outpatient setting. All the injection were performed by a single surgeon (FS) with level 3 experience according to Tang's criteria.^9^ After the injection the patients wore a bulky dressing for 3 days. 72 hours from the injection the patient came back to the outpatient clinic and we performed an injection of 5mm of lidocaine. After the onset of the local anaesthetic an extension force was applied on the affected digit to cause the rupture of the cord (Figure 2). The finger was immobilized in extension with a padded Zimmer splint 24 hours for 7 days and later during night for 30 days. We collected the following data before the injection: demographics, Tubiana's stage, MPJ contracture, PIPJ contracture, anamnesis for previous treatments, comorbidities, familiarity, the Disabilities of the Arm, Shoulder and Hand (DASH) score, complications, and recurrences. Complete correction was considered as residual contracture <5°, while recurrence of disease was defined as an increase in joint contracture to 20 degrees or more in the presence of a palpable cord at any time during the study.^10^ The outcomes were evaluated again at 3 months, 6 months, one year and two years. All the patients were evaluated by a single orthopaedic resident especially trained.

For the statistical analysis, a two-sample *t* test, chi-square test, and Fisher's exact test were used to test the significance of the cross-sectional differences between groups. Pearson's correlation coefficient was used to assess relationships between functional and radiographic out-comes. Age-adjusted univariate and forward stepwise multiple logistic regression analyses were used to determine whether explanatory variables were significantly associated with the recurrence of the pathology. Age-adjusted univariate and multiple forward stepwise linear regression analyses were also used to assess the association of independent variables with the 2 years DASH score. The explanatory variables including in the multivariate analysis were the following: age at operation, sex, Tubiana-stage, multiple cord (categorical: 0 = no; 1= yes), MPJ contracture (°), number of rays, previous surgery (categorical: 0 = no; 1= yes), diabetes (categorical: 0 = no; 1= yes), smoker (categorical: 0 = no; 1= yes), dialysis (categorical: 0 = no; 1= yes), familiarity (categorical: 0 = no; 1= yes), DASH T0, MPJ contracture at 2 years (°), PIPJ contracture (°) at 2 years, recurrence and skin breakage. Before construction of multivariate analysis models, an age-adjusted univariate analysis was performed. All explanatory variables that showed either an association or a trend toward an association (i.e. P<0.10) with the outcome of interest in the univariate analysis were included in our multiple regression models. In the multiple stepwise linear regression analysis, the total R^2^ for the model and the changes in R^2^ for the independent contribution of single explanatory variables were calculated to assess the percent of total variance in the outcome variable accounted for by the whole model and by single explanatory variables, respectively. A *p*-value of less than 0.005 was considered significant. The SPSS software program (SPSS, Inc., Chicago, IL, USA) was used for the database and statistics.

## RESULTS

From January 2018 through December 2019, we treated 66 consecutive patients with collagenase injections. The mean age of the patients was 64 years (range 33-84) and 57 patients were men (86,3%). Tubiana's stage I was present in 32 patients (48%), Tubiana's stage II in 32 patients (48%), while only 2 patients (3%) had Tubiana's stage III. Demographic and anamnestic details are resumed in Table 1. The mean pre-injection contracture was 34° (range 0°-90°) for MPJ and 31° (range 0°-90°) for PIPJ. At 3 months follow-up, complete correction (residual contracture 0–5°) in treated joints with baseline contracture ≥ 10° was observed in the 86% of MPJ and 26% of PIPJ. At 2-year follow-up, the mean contracture for the MPJ and PIPJ were 3° (range 0-24) and 14,5° (range 0°-60°), respectively. The mean pre-injection DASH score was 21.8 (range 0-80), while the mean DASH score at 2 years was 10,4 (range 0-51). (Table 2).

**Table 1:** Demographic and Anamnestic details.

Number of patients	66
**Gender**	
**male**	**57, (86,3%)**
female	9 (13,7%)
Mean age, years	64 (range 33-84)
**Digit involved**	
II	1 (1,5%)
III	7 (10,6%)
IV	23 (34,8%)
V	35 (53%)
**Severity (Tubiana's stage)**	
I	32 (48%)
II	32 (48%)
III	2 (3%)
**Comorbidity**	
Diabetes	14
Smokers	18
dialysis	4
Familiarity	13
**Previous treatment**	
Aponeurectomy	3
Shock waves	1

**Table 2:** Objective and subjective results.

	T0	3m	6m	1y	2y
Mean MPJ	34,15° (21)	2° (4,3)	2,2 °(4,6)	2,8° (5,4)	3° (5,8)
contracture (SD)					
Mean PIPJ	31° (29)	10° (11,4)	11,7° (14)	14,6° (17)	14,5° (17)
contracture (SD)					
Mean DASH (SD)	22 (17,3)	13,3 (16,7)	11 (14,6)	11,4 (13)	10,45 (13)

SD= standard deviation

The recurrence of the disease was observed in 23 patients (34.8%), all with PIP joint contracture. The main complication was skin breakage at time of the finger mobilization that occurred in 17 patients (25.7%), in 1 patient there was no rupture of the cord.

In our model of multiple linear regression analysis, the DASH score at final follow up was directly associated with the pre-injection DASH score (c=0.6; P<0.001) and the age at the time of injection (C=0.3; P=0.001). The pre-injection DASH score was by far the most influential predictor in this model, accounting for 71 % in variance of the outcome. The multivariate logistic regression analysis revealed that the pre-injection PIP contracture was the only predictor of recurrence of the pathology (odds ratio = 1.1; 95% confidence interval (CI) = 1.05 to 1.1; P<0.001).

At 2-year follow-up, the mean contracture for the MPJ and PIPJ were 3° (range 0-24) and 14,5° (range 0°-60°), respectively. Complete contracture correction, defined as residual contracture 0–5°, was achieved in the 75% of MPJ and in the 26% of the PIPJ.

## DISCUSSION

CCH injections are well supported in literature with positive contracture resolution.^11,12^ Our results correspond with the literature; one study^13^ of 57 patients reported contracture improvement of the MPJ from 54° to 9° and PIPJ from 30° to 16°, with complete resolution in 80% of MPJ and in 48% of PIPJ. Another study^14^ of 87 patients showed improvement from 39° to 14° in MPJ and from 54° to 32° in PIPJ, with a recurrence rate of 28.2% for MPJ and 62.1% for PIPJ. A third study^15^ of 77 patients, reported a mean contracture of the MPJ passing from 50° to 17° and of the PIPJ from 44° to 35.5°, with 4 recurrences within the first 6 weeks, and other 12 recurrences at 2-year follow-up (MPJ, 6; PIPJ, 6) reported. In our series, recurrence of the disease, defined as an increased contracture >20°, was observed only in the PIPJ, for a total of 23 patients (34.8%), and, moreover, we found that the only predictor of recurrence of the pathology was the pre-injection PIPJ contracture (odds ratio = 1.1; 95% confidence interval (CI) = 1.05 to 1.1; P<0.001). A higher recurrence rate for the PIP joints, regardless of treatment method, has been also reported by previous studies.^11,12,16^ Van Beeck et al.^14^ also stated that after the collagenase injection recurrence is more common for the PIPJ. One of the largest studies to assess the long-term efficacy of clostridium injection was the CORDLESS trial, which reported 5-year follow-up data for 623 joints.^11^ At 3-year follow-up, the recurrence rates were 16% for MPJ and 38% for PIPJ, while at 5-years follow-up increased to 39% and 66%, respectively. Most of the recurrences (75%) occurred in the first 3 years after treatment. Furthermore, the same authors^11^ compared collagenase injection with other techniques of treatment for DD, such as open or needle fasciotomy, and observed similar efficacy in contracture resolution and recurrence with lower complications rates for neurovascular injury and complex regional pain syndrome.

In our series the main complication was skin breakage at time of the finger mobilization that occurred in 17 patients (25.7%), while in 1 patient there was no rupture of the cord. The occurrence of skin tears was recorded in previous studies,^8,17^ as well as cord rupture failure.^18^ Some studies^18,19^ consider skin tears an expected mechanical consequence due to the mechanism of correction rather than a complication, especially for the more severe contractures, demonstrating an increasing risk of a skin tear with increasing contracture severity.^20^ Anyway, in our series they all healed quickly without infection, also as previously reported.^17^

We used the DASH questionnaire as subjective outcomes tool and the mean score at the latest follow-up was 10.4. The DASH score at final follow up was directly associated with the pre-injection DASH score (c=0.6; P<0.001) and the age at the time of injection (C=0.3; P=0.001). The pre-injection DASH score was by far the most influential predictor in this model, accounting for 71% in variance of the outcome. Lauritzon et al^13^ observed that the changes in joint contracture and patient satisfaction were associated to the entity of DASH improvement. To date, there is debate about the utility of any patient rated outcome measure for Dupuytren's disease. Even if some authors have tried to validate the DASH questionnaire for Dupuytren's disease, it has not been found to be particularly reflective of clinical changes.^21^ In a recent review^8^ the authors observed that only few studies used subjective outcome tools and concluded that future studies should concentrate on patient-related outcomes.

The strengths of our study include its prospective design and the use of reliable subjective and objective clinical measurements to assess the outcomes.

The study has some limitations: first the short follow up could underestimate the percentage of recurrence.

## CONCLUSIONS

Serious adverse effects with CCH injections are uncommon and less frequent compared to the rate of major complication occurred after surgical fasciectomy. CCH injection remains a consistent option in the treatment of DD; the withdrawal of the drug from European market deprives surgeons and patients of a low invasiveness and safety tool which has changed the treatment of DD.
